# What we talk about when we talk about the default mode network

**DOI:** 10.3389/fnhum.2014.00619

**Published:** 2014-08-25

**Authors:** Felicity Callard, Daniel S. Margulies

**Affiliations:** ^1^Centre for Medical Humanities and Department of Geography, Durham UniversityDurham, UK; ^2^Max Planck Research Group for Neuroanatomy and Connectivity, Max Planck Institute for Human Cognitive and Brain SciencesLeipzig, Germany

**Keywords:** functional connectivity, neuroanatomy, resting state, fMRI, history of cognitive neuroscience, mind wandering

## Abstract

The default mode network (DMN) has been widely defined as a set of brain regions that are engaged when people are in a “resting state” (left to themselves in a scanner, with no explicit task instruction). The network emerged as a scientific object in the early twenty-first century, and in just over a decade has become the focus of intense empirical and conceptual neuroscientific inquiry. In this Perspective, we contribute to the work of critical neuroscience by providing brief reflections on the birth, working life, and future of the DMN. We consider: how the DMN emerged through the convergence of distinct lines of scientific investigation; controversies surrounding the definition, function and localization of the DMN; and the lines of interdisciplinary investigation that the DMN has helped to enable. We conclude by arguing that one of the most pressing issues in the field in 2014 is to understand how the mechanisms of thought are related to the function of brain dynamics. While the DMN has been central in allowing the field to reach this point, it is not inevitable that the DMN itself will remain at the heart of future investigations of this complex problem.

## Introduction

**Figure 1:**
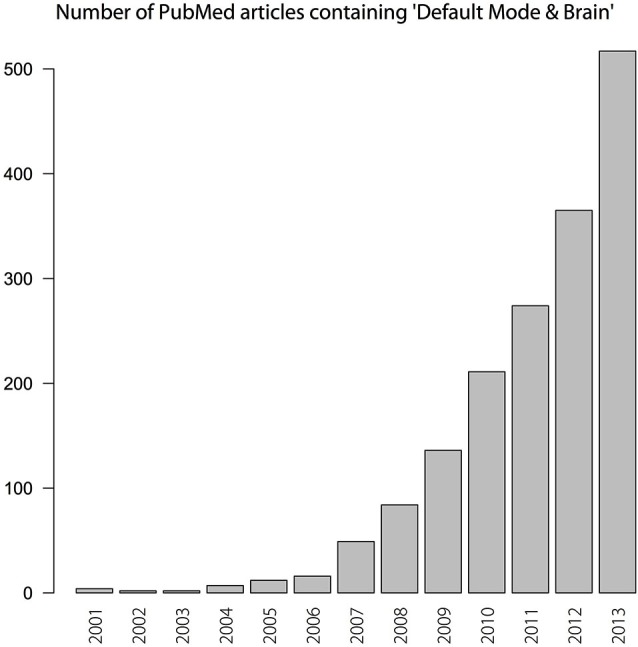
**Number of PubMed articles that report “Default Mode” or “Default Mode Network” and “Brain” from 2001–2013**.

The *default mode network* (DMN)—at times termed the *default network* (e.g., Buckner et al., 2008)—came to prominence in cognitive neuroscience as a set of brain regions that are engaged when people are in a “resting state” (left to themselves in a scanner, with no explicit task instruction). The DMN is about a decade old: it emerged in the early part of the twenty-first century—although exact dates of birth are a vexed topic when one is talking about a scientific object. Since its emergence, interest in the DMN has been intense and growing (see Figure 1).

If one of the tasks of “critical neuroscience” is to investigate “the history of concepts, practices and objects of scientific inquiry” (Slaby and Choudhury, 2012), then in this Perspective we contribute to critical neuroscience by reflecting on the birth, working life, and potential future of the scientific object that is the DMN. We are indebted to several historians of science, particularly Daston (2000) and Rheinberger (1997), who have convincingly demonstrated how scientific objects—objects that are subject, in particular times and places, to intense interest and investigation by working scientists—should be thought of as “simultaneously real *and* historical” (Daston, 2000, p. 3). On such a model, scientific objects emerge at the intersection point of various practices, scientific apparatuses, conceptual frameworks and techniques, and cannot be understood outside of them. Just as scientific objects emerge, so can and do they at times decay or disappear: in other words, the aura of any scientific object is always colored by its potential for obsolescence.

## Convergence, transformation and controversy

The DMN emerged through the coming together of two distinct lines of enquiry, which we have characterized as the neurophysiological and the neuropsychological (Callard and Margulies, 2011). The unified field currently known as “resting-state fMRI research”—a field intimately associated with the operations of the DMN—was derived from two distinct lineages: Biswal et al.’s research in the mid-1990s on functional connectivity during the resting-state (Biswal et al., 1995; Biswal, 2012), and Raichle and colleagues’ observation of a set of regions that were consistently higher in activity during the resting, baseline task-state (Buckner, 2012; Snyder and Raichle, 2012).

Notably, the DMN emerged through the work of contrasting it with that which it was considered, ontologically, *not* to be. Initially, the task contrast was used to define the regions of the DMN, which included those that were deactivated during performance of a task when compared to the resting, baseline condition (Shulman et al., 1997b; Gusnard et al., 2001; Raichle et al., 2001). This task-centric definition was then expanded in a foundational study by Greicius et al. (2003), which linked Biswal’s resting-state functional connectivity methodology (e.g., Biswal et al., 1995) to the default mode hypothesis (Gusnard et al., 2001), and coined the term “DMN”. The DMN emerged, then, in juxtaposition with, if not in explicit opposition to, externally-focused tasks. Greicius et al.’s (2003) article, which is commonly thought to have launched the DMN, noted that certain brain regions “consistently show greater activity during resting states *than during cognitive tasks*” [italics added]. This bifurcatory framework—between (particular kinds of) cognitive tasks and psychological operations associated with the DMN—has persisted, even as it is now frequently attenuated by greater acknowledgement of the undeniable fact that cognitive work should not be restricted to externally-focused tasks. The Cognitive Atlas currently defines the DMN as “an organized spontaneous network of neural activity *that is modulated during attention-demanding cognition*” [italics added], which is characterized by “spontaneous BOLD signal fluctuations which tend to inversely correlate with fluctuations in other networks, including those that subserve arousal, attention, perception, and working memory”.^1^ That this bifurcation has structured discussions of the DMN from its birth has not only shaped what we *do* talk about when we talk about the DMN (e.g., lapses or breaks in attention) but also what we *do* not—or only rarely—talk about (e.g., the potential ways in which DMN activity might well be associated with attention—though attention conceptualized in ways different from the standard model of external task-based attention; see also Callard et al., 2012).

Expanding the terrain of interest from task to rest, from activation to connectivity, from a brain state (the “default mode”) to a brain network (the “DMN”), opened up various possibilities for describing what has been imagined as the same scientific object. The DMN gained solidity through the conjunction of different kinds of experimental apparatus, various experimental data, different sets of disciplinary expertise, and different scientific preoccupations. Methods derived from Biswal’s (1995) resting-state functional connectivity (essentially originating in the question of the constituent components of fMRI signal fluctuations), when placed alongside Raichle et al.’s (2001) questions regarding the physiological baseline of blood oxygen-level dependent (BOLD) contrast, provided multiple ways to derive what is commonly assumed to be the same structure. But what we want really to bring to visibility is how the different methods used to probe and delineate what has come to be called the DMN have constituted the network in different ways:

- Shulman et al. (1997a) reanalyzed nine PET studies of visual processing.
- Binder et al. (1999) measured brain activation during rest using several contrasting activation states (including tone monitoring and semantic retrieval).
- Andreasen et al. (1995)—which, while published in the 1990s, really came only retrospectively to be folded into the history of the “discovery” of the DMN (e.g., see Binder, 2012; Buckner, 2012)—contrasted two different kinds of memory (what they termed “focused episodic memory” and “random episodic memory” [or Random Episodic Silent Thinking: REST]). They conceptualized *both* in terms of activations.
- Studies using measures from graph theory (which represents the topology of complex systems in terms of elements (nodes) and their mutual relationships (edges)), such as centrality, have described the core regions of the DMN independently of psychological categories (e.g., van den Heuvel and Sporns, 2013; Sporns, 2014).

We provide these examples for two reasons. First, the DMN emerged into the light from the dark underside of cognition through a *variety* of contrasted conditions. That the network became a positive entity via a serendipitous discovery (see Buckner, 2012), rather than as a hypothesized entity, meant that it was able to remain ontologically capacious. A number of answers could therefore be given to the questions: what exactly is this (initially under-described and under-defined) entity? What does it do—both neuroanatomically and psychologically? How does it differ from the various functions and activities with which it has been contrasted? The historian of science Cornelius Borck provides a fascinating historical comparison here, in his analysis of physiologist Edgar Adrian’s findings regarding intrinsic ganglionic activity. These findings pushed Adrian to replicate Hans Berger’s findings regarding “brain waves” and thereby challenged Adrian’s own formulations regarding the universal code of the nervous system (Borck, 2008). The intrinsic activity of the brain, which, captured by the electrocardiograph as a stable, rhythmic oscillation of approximately 10 Hz, was shown to be disturbed by the work of mental arithmetic. That both kinds of intrinsic activity (the brain rhythms captured by the EEG, and the slow cortical fluctuations revealed via resting-state fMRI) were subsequently tied to a variety of high-level psychological constructs perhaps points to an enduring urge to endow the working of the brain in its “idle” or “default” state with a dense psychological hermeneutics.^2^

Second, the concept of a network—which describes any interconnected arrangement or topology of interrelated nodes—has, itself, a complex history in the life sciences and social sciences (e.g., Latour, 1996; Offner, 1999), as well as more specifically in the neurosciences. Current uses of “network” in graph theory models have interesting intersections with, but cannot be assumed wholly to overlap with, earlier models of neural networks that were indebted to the foundational research of those such as Bain (1873) and Hebb (1949) (which are still influential in shaping understandings of what a brain network is). One of the strengths of the umbrella term “network” is that it is able to draw together a variety of models, understandings and modes of conceptualizing interrelated entities—which themselves cross the terrains of the anatomical, functional and psychological. While the “DMN” is commonly assumed to have an undeniable reality because different methods of investigation have converged on what is assumed to be the same ontological entity (e.g., Shulman’s, Binder’s, Andreasen’s, and van den Heuvel and Sporns’ studies mentioned above are commonly understood as uncovering *the same thing*), it remains unclear how exactly the diverse anatomical, functional and psychological models of the outline and activities of this entity relate to, and should best be sutured to one another.

One notable example comprises the varied descriptions of the network’s anatomical components. Controversies surrounding the localization of various cortical regions are not uncommon in functional neuroanatomy, especially when the areas of interest shift beyond well-characterized functions of sensorimotor cortex. However, the challenge of defining the anatomical components of the DMN is further confounded by its multiple operational definitions, each suggesting subtle spatial variations from a core set of cortical regions along the anterior and posterior medial wall. The notion of *subsystems* within the DMN has been proposed to better describe the multiple network variants that spatially overlap with the key medial structures (Buckner et al., 2008; Andrews-Hanna et al., 2010). The challenge of spatiotemporally localizing the DMN is further complicated by observations that the synchronous activity of its various regions, the defining feature of functional connectivity, is modulated dynamically (Chang and Glover, 2010). While the DMN resides at the nexus of multiple converging lines of research, upon close inspection from any one angle, its features appear progressively less well defined.

## To the core of the human animal? The function of the DMN

Delineations of the psychological functions of the DMN show how its semantic capaciousness has resulted in multiple operational definitions, some of which have entailed perhaps over hasty normative judgments about the function of the DMN in relation to the kind of human animals that we are assumed to be.

From the start, the DMN was associated with high-level, complex cognitive processes regarded by many as lying at the heart of what makes the human animal “human”. For example, Gusnard et al. (2001) linked the default mode to self-referential mental activity: indeed, the authors argued that the “default state of the brain instantiates functions that are integral to the self”. Continued research has further established the self-referential function implicating the DMN (D’Argembeau et al., 2005; Buckner and Carroll, 2007; Salomon et al., 2009, 2014). Greicius et al. (2003) argued in their seminal 2003 paper that mapping the DMN “may provide insight into the neural underpinnings of a critical but poorly understood component of human consciousness variably referred to as “a conscious resting state”, “stimulus-independent thought”, or a “default mode of brain”. Mazoyer et al. (2001) stated that “A large part of our daily mental activities are internalized, *id est* performed without input or motor output, and not goal directed”—and raised the possibility that this “particular state of consciousness” is associated both with the monitoring of “somesthesic and vegetative information, … and experiencing association of *free thoughts* that deal with the recollection of past experiences, inner speech, mental images, emotions, planning of future activities, etc.” Andreasen et al. (1995) understood their study to be “explor[ing] the neural circuitry of the activities of mind as well as brain, especially those that impinge on important human characteristics, such as the capacity for consciousness, self awareness, and creativity”. fMRI activations associated with the resting state were, from this early work onwards (e.g., Andreasen et al., 1995; Binder et al., 1999), considered candidates for higher psychological processes rather than being regarded as solely associated with physiological functions. In this regard, the ground had been laid by earlier research by David Ingvar on regional cerebral blood flow (rCBF), using the 133 Xenon clearance technique. Ingvar, in documenting hyperfrontal distribution of the “cerebral gray matter flow in resting wakefulness”, argued that such frontal activity “implie[d] … a temporal sequence of thoughts with a goal direction”—in short, that the brain was “automatically busy with extrapolation of future events … in order to be ready for what may happen” (Ingvar, 1979).

Virtually all the constructs mentioned in the paragraph above operate at a high level of abstraction (e.g., “self”, “free thought”, “self awareness”, “creativity”): it might almost be said that every high-level psychological construct was imagined as being potentially associated with this network’s operations. Considering that the DMN’s regions constitute a substantial portion of associative cortex, implication of the DMN across the breadth of higher function may not be inaccurate, but if its functions account for everything, the research challenge is shifted to understanding the workings of its subcomponents.

More recently, investigation of the psychological functions of the DMN has been increasingly enabled through one (equally high-level) construct: that of “mind-wandering”. In Callard et al. (2013), we provided a conceptual and empirical analysis not only of an increasingly intimate association (from 2007 onwards) between research on mind-wandering and research on the DMN, but of how much of this research has been “negatively” driven by characterizing the network’s psychological activity as a “break” or “lapse”, or through explicitly contrasting it with some of the key constructs employed within cognitive psychology (e.g., attention, memory) (Weissman et al., 2006). While the close association between mind-wandering research and DMN research has been enormously productive for both arenas of inquiry, it has likely led to too tight an association between mind-wandering and the so-called “task-negative” network. To imagine that such a complex phenomenological experience as described by the term “mind-wandering” might be subserved by one brain network alone runs the risk of restricting investigation of more heterogeneous forms of spontaneous cognition. This one-to-one mapping makes it harder to investigate the complex inter-relations between a number of networks that might well contribute to these heterogeneous psychological states. One key question for the field, then, is what consequences follow from a tight interlocking of (one) mental experience (mind-wandering) and (one) brain network (DMN). Other ways of envisaging relations between psychological processes and brain dynamics might well emerge in its place.

Secondly, the tendency to install characteristics that lie at the core of human subjectivity (e.g., creativity, free thoughts, etc.) within the DMN has gone hand in hand with a tendency to judge the kind of human animal that we are. Killingsworth and Gilbert (2010), for example, argue that “a human mind is a wandering mind, and a wandering mind is an unhappy mind. The ability to think about what is not happening is a cognitive achievement that comes at an emotional cost.” The strong ontological claim about the unhappy human mind is hung on one, necessarily limited, empirical study. Such strong normative claims, in relation to a youthful field in which so much is still unclear, are striking and, we contend, likely to be premature.

## Whither the DMN?

The DMN has been remarkably productive in bringing hitherto marginalized fields and methods inside the perimeters of cognitive neuroscience—and, through such incursions, sparked new lines of conceptual and methodological inquiry. Topics such as mind-wandering, previously considered largely beyond the purview of cognitive psychology, have emerged as heated areas of research (Callard et al., 2013). Neuropsychoanalytic researchers have found the DMN to be a rich concept through which to advance formulations about psychic energy (Carhart-Harris and Friston, 2010), psychodynamic concepts of self in relation to objects (Northoff, 2011) and fantasy (Zellner, 2013). There is currently methodological interest in using a variety of tools, including introspection, descriptive experience sampling, and free association (many of which are marginalized in cognitive psychology) to access the complex shapes of “inner experience” characteristic of the default mode (e.g., Fell, 2013). The DMN, in collaboration with other resting-state networks, has opened the possibility for network-level interpretations of brain function.

The DMN has had great success in generating fertile tracks for scientific inquiry. As Daston has argued, the creation and solidification of a scientific object, via the drawing together of heterogeneous findings and phenomena, can help generate a wide variety of novel empirical and theoretical formulations (Daston, 2000, p. 5). Rheinberger has demonstrated how productive “imprecise” scientific objects with fuzzy boundaries can be. Such objects, with their “characteristic, irreducible vagueness” are *necessarily* precarious: they are “absent in their experimental presence”, since they are characterized precisely by that which scientists *do not yet know* (Rheinberger, 1997, p. 28).

The DMN has accomplished its initial mission of making it possible to ask questions about baseline brain activity within cognitive neuroscience. In this respect, it is worth considering whether the network plays a similar role to that played by mirror neurons, about which Ramachandran (2000) argued, in optimistic vein: “mirror neurons will do for psychology what DNA did for biology: they will provide a unifying framework and help explain a host of mental abilities that have hitherto remained mysterious and inaccessible to experiments”. The high level abstraction of the DMN (due partly to the broad definition of its function) allows for sharing between several diverse research communities with different understandings of the meanings and characteristics of “default”, “mode”, and “network”.

In 2014, as the concept of the DMN reaches adolescence, one question to consider is whether—and when—it might be transformed into or translated into other scientific objects. The first decade of the DMN’s life could be characterized through two, overlapping phases:

- Phase 1: Exploration of various functions involved in the default mode (the era of the discovery of intrinsic brain activity).
- Phase 2: Exploration of the function of the DMN *qua* network (through the tools of functional connectivity).

We contend that the central task of Phase 3, whose time is now, comprises the exploration of *thought qua thought* and its relation to intrinsic brain dynamics. Building on earlier work that describes the DMN as the primary seat of mind wandering, the current state of research on spontaneous cognition extends the terrain of inquiry, pointing to the involvement of regions both within and outside of the DMN (Andrews-Hanna et al., 2014). As the research agenda turns to the question of spontaneous thought itself, rather than specifically the function of the DMN, the path of inquiry that the DMN has enabled is, perhaps, on the way to making its key role unnecessary.

The DMN might, then, at a certain point, either shift into becoming an entity that is taken for granted in everyday life (but one that is not the focus of intense scientific investigation) or might disintegrate or even disappear. Although it has enabled new fields to arise around critical research questions, we should not take for granted that it will be the DMN as a scientific object that will be most critical in effecting progress in understanding the relationship between thought and brain dynamics.

## Conflict of interest statement

The authors declare that the research was conducted in the absence of any commercial or financial relationships that could be construed as a potential conflict of interest.

## Footnotes

^1^http://www.cognitiveatlas.org/concept/default_mode_network [accessed 2 May 2014].

^2^We thank the reviewer Cornelius Borck for drawing out the fascinating historical comparisons with EEG, and the work of Berger and Adrian—and not least for pointing out how in both instances a still vague scientific object was linked to high-level psychological constructs.

## References

[B1] Andreasen N. C. O’Leary D. S. Cizadlo T. Arndt S. Rezai K. Watkins G. L. (1995). Remembering the past: two facets of episodic memory explored with positron emission tomography. Am. J. Psychiatry 152, 1576–1585 7485619 10.1176/ajp.152.11.1576

[B2] Andrews-Hanna J. R. Reidler J. S. Sepulcre J. Poulin R. Buckner R. L. (2010). Functional-anatomic fractionation of the brain’s default network. Neuron 65, 550–562 10.1016/j.neuron.2010.02.005 20188659 PMC2848443

[B3] Andrews-Hanna J. R. Smallwood J. Spreng R. N. (2014). The default network and self-generated thought: component processes, dynamic control and clinical relevance. Ann. N Y Acad. Sci. 1316, 29–52 10.1111/nyas.12360 24502540 PMC4039623

[B4] Bain A. (1873). Mind and Body: The Theories of their Relation. 2nd Edn. London: Henry S. King & Co

[B5] Binder J. R. (2012). Task-induced deactivation and the “resting” state. Neuroimage 62, 1086–1091 10.1016/j.neuroimage.2011.09.026 21979380 PMC3389183

[B6] Binder J. R. Frost J. A. Hammeke T. A. Bellgowan P. S. Rao S. M. Cox R. W. (1999). Conceptual processing during the conscious resting state. A functional MRI study. J. Cogn. Neurosci. 11, 80–95 10.1162/089892999563265 9950716

[B8] Biswal B. B. (2012). Resting state fMRI: a personal history. Neuroimage 62, 938–944 10.1016/j.neuroimage.2012.01.090 22326802 PMC12911935

[B7] Biswal B. Yetkin F. Z. Haughton V. M. Hyde J. S. (1995). Functional connectivity in the motor cortex of resting human brain using echo-planar MRI. Magn. Reson. Med. 34, 537–541 10.1002/mrm.1910340409 8524021

[B9] Borck C. (2008). Recording the brain at work: the visible, the readable and the invisible in electroencephalography. J. Hist. Neurosci. 17, 367–379 10.1080/09647040701348332 18629702

[B10] Buckner R. L. (2012). The serendipitous discovery of the brain’s default network. Neuroimage 62, 1137–1145 10.1016/j.neuroimage.2011.10.035 22037421

[B12] Buckner R. L. Andrews-Hanna J. R. Schacter D. L. (2008). The brain’s default network: anatomy, function and relevance to disease. Ann. N Y Acad. Sci. 1124, 1–38 10.1196/annals.1440.011 18400922

[B11] Buckner R. L. Carroll D. C. (2007). Self-projection and the brain. Trends Cogn. Sci. 11, 49–57 10.1016/j.tics.2006.11.004 17188554

[B13] Callard F. Margulies D. S. (2011). The subject at rest: novel conceptualizations of self and brain from cognitive neuroscience’s study of the “resting state”. Subjectivity 4, 227–257 10.1057/sub.2011.11

[B15] Callard F. Smallwood J. Golchert J. Margulies D. S. (2013). The era of the wandering mind? Twenty-first century research on self-generated mental activity. Front. Psychol. 4:891 10.3389/fpsyg.2013.00891 24391606 PMC3866909

[B14] Callard F. Smallwood J. Margulies D. S. (2012). Default positions: how neuroscience’s historical legacy has hampered investigation of the resting mind. Front. Psychol. 3:321 10.3389/fpsyg.2012.00321 22973252 PMC3437462

[B16] Carhart-Harris R. L. Friston K. J. (2010). The default-mode, ego-functions and free-energy: a neurobiological account of Freudian ideas. Brain 133, 1265–1283 10.1093/brain/awq010 20194141 PMC2850580

[B17] Chang C. Glover G. H. (2010). Time-frequency dynamics of resting-state brain connectivity measured with fMRI. Neuroimage 50, 81–98 10.1016/j.neuroimage.2009.12.011 20006716 PMC2827259

[B19] D’Argembeau A. Collette F. Van der Linden M. Laureys S. Del Fiore G. Degueldre C. (2005). Self-referential reflective activity and its relationship with rest: a PET study. Neuroimage 25, 616–624 10.1016/j.neuroimage.2004.11.048 15784441

[B20] Daston L. (2000). “The coming into being of scientific objects,” in Biographies of Scientific Objects, ed Daston L. (Chicago: University of Chicago Press), 1–14

[B21] Fell J. (2013). Unraveling inner experiences during resting state. Front. Hum. Neurosci. 7:409 10.3389/fnhum.2013.00409 23908620 PMC3725401

[B22] Greicius M. D. Krasnow B. Reiss A. L. Menon V. (2003). Functional connectivity in the resting brain: a network analysis of the default mode hypothesis. Proc. Natl. Acad. Sci. U S A 100, 253–258 10.1073/pnas.0135058100 12506194 PMC140943

[B23] Gusnard D. A. Raichle M. E. Raichle M. E. (2001). Searching for a baseline: functional imaging and the resting human brain. Nat. Rev. Neurosci. 2, 685–694 10.1038/35094500 11584306

[B24] Hebb D. O. (1949). The Organization of Behavior. A Neuropsychological Theory. New York: John Wiley and Sons; London: Chapman and Hall

[B25] Ingvar D. H. (1979). “Hyperfrontal” distribution of the cerebral grey matter flow in resting wakefulness; on the functional anatomy of the conscious state. Acta Neurol. Scand. 60, 12–25 10.1111/j.1600-0404.1979.tb02947.x 495039

[B26] Killingsworth M. A. Gilbert D. T. (2010). A wandering mind is an unhappy mind. Science 330, 932 10.1126/science.1192439 21071660

[B27] Latour B. (1996). On actor-network theory. A few clarifications plus more than a few complications. Soz. Welt 47, 369–381

[B28] Mazoyer B. Zago L. Mellet E. Bricogne S. Etard O. Houde O. (2001). Cortical networks for working memory and executive functions sustain the conscious resting state in man. Brain Res. Bull. 54, 287–298 10.1016/s0361-9230(00)00437-8 11287133

[B29] Northoff G. (2011). Self and brain: what is self-related processing? Trends Cogn. Sci. 15, 186–187 10.1016/j.tics.2011.03.001 21458358

[B30] Offner J.-M. (1999). “Are there such things as small networks?,” in The Governance of Large Technical Systems, ed Coutard O. (London and New York: Routledge), 217–238

[B31] Raichle M. E. MacLeod A. M. Snyder A. Z. Powers W. J. Gusnard D. A. Shulman G. L. (2001). A default mode of brain function. Proc. Natl. Acad. Sci. U S A 98, 676–682 10.1073/pnas.98.2.676 11209064 PMC14647

[B32] Ramachandran V. S. (2000). Mirror neurons and imitation learning as the driving force behind “the great leap forward” in human evolution. Edge. Available online at: http://www.edge.org/3rd_culture/ramachandran/ramachandran_p1.html Accessed 2 May 2014.

[B33] Rheinberger H.-J. (1997). Toward a History of Epistemic Things: Synthesizing Proteins in the Test Tube. Stanford, California: Stanford University Press

[B34] Salomon R. Levy D. R. Malach R. (2014). Deconstructing the default: cortical subdivision of the default mode/intrinsic system during self-related processing. Hum. Brain Mapp. 35, 1491–1502 10.1002/hbm.22268 23568328 PMC6869590

[B35] Salomon R. Malach R. Lamy D. (2009). Involvement of the intrinsic/default system in movement-related self recognition. PLoS One 4:e7527 10.1371/journal.pone.0007527 19844584 PMC2760765

[B36] Shulman G. L. Corbetta M. Buckner R. L. Fiez J. A. Miezin F. M. Raichle M. E. (1997a). Common blood flow changes across visual tasks: I. Increases in subcortical structures and cerebellum but not in nonvisual cortex. J. Cogn. Neurosci. 9, 624–647 10.1162/jocn.1997.9.5.624 23965121

[B37] Shulman G. L. Fiez J. A. Corbetta M. Buckner R. L. Miezin F. M. Raichle M. E. (1997b). Common blood flow changes across visual tasks: II. Decreases in cerebral cortex. J. Cogn. Neurosci. 9, 648–663 10.1162/jocn.1997.9.5.648 23965122

[B18] Slaby J. Choudhury S. (2012). “Proposal for a critical neuroscience,” in Critical Neuroscience: A Handbook of the Social and Cultural Contexts of Neuroscience, eds Choudhury S. Slaby J. (Chichester, West Sussex: Wiley-Blackwell).

[B38] Snyder A. Z. Raichle M. E. (2012). A brief history of the resting state: the Washington University perspective. Neuroimage 62, 902–910 10.1016/j.neuroimage.2012.01.044 22266172 PMC3342417

[B39] Sporns O. (2014). Contributions and challenges for network models in cognitive neuroscience. Nat. Neurosci. 17, 652–660 10.1038/nn.3690 24686784

[B40] van den Heuvel M. P. Sporns O. (2013). Network hubs in the human brain. Trends Cogn. Sci. 17, 683–696 10.1016/j.tics.2013.09.012 24231140

[B41] Weissman D. H. Roberts K. C. Visscher K. M. Woldorff M. G. (2006). The neural bases of momentary lapses in attention. Nat. Neurosci. 9, 971–978 10.1038/nn1727 16767087

[B42] Zellner M. R. (2013). Dreaming and the default mode network. Contemp. Psychoanal. 49, 226–232 10.1080/00107530.2013.10746548

